# Functional Germline DNA Repair Mutations as Predictors of Acute Radiodermatitis in Breast Cancer

**DOI:** 10.3390/diagnostics16060833

**Published:** 2026-03-11

**Authors:** Andreea Cătană, Andrada-Adelaida Belbe, Daniela Laura Martin, Horațiu Ciliboaie, Mariela Sanda Militaru, Irina Ioana Iordănescu, Patriciu Achimaș-Cadariu, Lorin-Manuel Pîrlog

**Affiliations:** 1 Department of Molecular Sciences, Faculty of Medicine, “Iuliu Hațieganu” University of Medicine and Pharmacy, 400012 Cluj-Napoca, Romania; catanaandreea@elearn.umfcluj.ro (A.C.); sanda.militaru@umfcluj.ro (M.S.M.); lorin.pirlog@gmail.com (L.-M.P.); 2 Department of Oncogenetics, “Prof. Dr. I. Chiricuță” Institute of Oncology, 400015 Cluj-Napoca, Romania; 3 Regional Laboratory Cluj-Napoca, Department of Medical Genetics, Regina Maria Health Private Network, 400363 Cluj-Napoca, Romania; 4 Department of Radiotherapy, “Prof. Dr. I. Chiricuță” Institute of Oncology, 400015 Cluj-Napoca, Romania; drdanielamartin@yahoo.com (D.L.M.); horatiu.ciliboaie@gmail.com (H.C.); 5 Genetic Centre Laboratory, Department of Medical Genetics, Regina Maria Health Private Network, 011376 Bucharest, Romania; irina.iordanescu@reginamaria.ro; 6 1st Department of Oncologic Surgery, “Prof. Dr. I. Chiricuță” Institute of Oncology, 400015 Cluj-Napoca, Romania; pachimas@umfcluj.ro

**Keywords:** acute radiodermatitis, breast cancer, DNA repair pathways, homologous recombination repair, Fanconi anemia pathway, DNA damage response, tumor suppressor genes, germline mutations, radiosensitivity, hypofractionated IMRT

## Abstract

**Background/Objectives**: Acute radiotherapy-induced skin toxicity is a common complication in breast cancer treatment, with marked interindividual variability not fully explained by clinical factors. This study investigated the contribution of germline mutations in DNA repair and tumor suppressor genes to acute radiodermatitis in a homogeneous cohort treated with hypofractionated intensity-modulated radiotherapy with inverse planning, with adjustment for potential lifestyle confounders. **Methods**: Mutations were grouped into four functional categories: homologous recombination repair (HRR), Fanconi anemia (FA), DNA damage response (DDR), and tumor suppressor (TS) genes. Ordinal logistic regression models adjusted for clinical covariates evaluated pooled and functional category-specific mutation effects. **Results**: Overall, any mutation significantly increased the risk of higher-grade acute radiodermatitis (OR = 2.24, *p* = 0.003), an effect driven primarily by HRR and FA mutations, as exclusion of these mutations rendered the association non-significant (OR = 1.785, *p* = 0.064). Functional category-based analyses showed that HRR (OR = 2.60, *p* = 0.002) and FA (OR = 2.62, *p* = 0.002) mutations were the strongest predictors, reflecting overlapping roles in double-strand break and interstrand crosslink repair. DDR and TS mutations showed no significant effect. **Conclusions**: These results highlight the key role of high-fidelity DNA repair in normal tissue radiosensitivity and demonstrate that functional genetic stratification has diagnostic value as a pre-treatment predictive biomarker framework, enabling identification of patients at increased risk of acute skin toxicity and supporting personalized radiotherapy planning.

## 1. Introduction

Adjuvant radiotherapy constitutes a cornerstone of contemporary breast cancer management, conferring substantial reductions in locoregional recurrence and enhancing overall survival outcomes. The implementation of advanced radiation delivery modalities, including intensity-modulated radiotherapy with inverse planning (IMRT-IP) and hypofractionated dose schedules, has enhanced dosimetric precision while minimizing exposure to adjacent normal tissues [1,2]. Notwithstanding these technological refinements, acute radiation-induced dermatitis remains a prevalent complication that may compromise treatment adherence, diminish health-related quality of life, and necessitate therapeutic interruption in select cases [1,3].

Substantial interindividual heterogeneity in acute radiodermatitis severity has been documented among breast cancer patients receiving ostensibly equivalent radiation doses and treatment modalities. While cutaneous radiation reactions occur in many treated individuals, the spectrum of toxicity ranges from transient erythema to confluent moist desquamation that can impair functional capacity and necessitate treatment modification [1]. Established patient-specific and treatment-related determinants—including body mass index, tobacco use, breast volume, antecedent systemic therapy, and dosimetric parameters—have been implicated in modulating toxicity risk. Nevertheless, these conventional clinical and behavioral risk factors do not fully explain the substantial variance in normal tissue radioresponse observed in clinical practice [4,5].

At the molecular level, radiation-induced cutaneous injury arises from intricate interactions among DNA damage induction, pro-inflammatory cytokine cascades, microvascular endothelial dysfunction, and depletion of proliferative basal keratinocyte populations. Interindividual variation in DNA damage recognition efficiency, repair pathway fidelity, and downstream cellular stress responses may critically modulate tissue recovery kinetics following ionizing radiation exposure. These mechanistic insights underscore the existence of an intrinsic biological substrate governing individual radiosensitivity, thereby providing a compelling rationale for systematic investigation of genetic determinants of acute radiotherapy-induced dermatitis independent of conventional clinical and behavioral risk factors [6].

Ionizing radiation induces a heterogeneous spectrum of DNA lesions, most critically DNA double-strand breaks (DSBs), the accurate detection and repair of which are essential for normal tissue homeostasis following therapeutic irradiation. Germline or somatic alterations within DNA damage response (DDR) and repair pathways may compromise these protective mechanisms, thereby augmenting susceptibility to radiation-induced injury in non-malignant tissues. While rare monogenic DNA repair deficiency syndromes are well-established paradigms of constitutional radiosensitivity, the contribution of more prevalent pathogenic or likely pathogenic germline variants to acute radiotherapy-associated toxicity remains inadequately elucidated [7,8].

Previous studies examining genetic predictors of radiodermatitis have produced inconsistent results, often limited by heterogeneous treatment techniques, small sample sizes, or gene-centric analytical approaches [5,9]. Grouping genetic alterations according to their functional roles within coordinated DNA repair pathways may provide a more biologically informative framework for understanding normal tissue radiosensitivity. Pathways involved in homologous recombination and Fanconi-mediated DNA repair are critical for resolving complex radiation-induced DNA damage and may be especially relevant in rapidly proliferating tissues such as the epidermis [10,11,12,13].

Because radiodermatitis represents a radiation-induced toxicity affecting normal skin tissue rather than tumor cells, host constitutional genetic background is biologically more relevant than tumor-specific genomic alterations for predicting individual radiosensitivity. Germline DNA repair variants are present in all somatic cells, including irradiated healthy skin, and therefore may directly modulate normal tissue response to radiation-induced DNA damage. In contrast, somatic mutations are confined to tumor tissue and reflect dynamic, heterogeneous processes of clonal evolution that primarily influence tumor behavior and treatment response. Given our objective to identify stable, pre-treatment biomarkers of normal tissue toxicity, we focused on germline alterations. In addition, our analysis accounted for patient-intrinsic factors, treatment parameters, and lifestyle-related variables as relevant confounders. Moreover, while somatic genomic predictors of tumor response have previously been explored, data regarding germline mutations and radiodermatitis remain limited and inconsistent, underscoring the need for further investigation in this area.

In the present investigation, we systematically evaluated the association between germline mutation status and acute radiation-induced dermatitis severity in a clinically homogeneous cohort of breast cancer patients treated with adjuvant hypofractionated IMRT-IP. Employing genetic functional category-based stratification and multivariable ordinal logistic regression models adjusted for established clinical and lifestyle confounders, we aimed to identify diagnostically informative functional genetic categories that predict elevated risk of higher-grade acute radiodermatitis and may serve as molecular biomarkers for pre-treatment patient stratification. The findings presented herein advance mechanistic understanding of normal tissue radiosensitivity and support the integration of genomic diagnostics into clinical decision-making frameworks for personalized radiotherapy planning and toxicity mitigation.

## 2. Materials and Methods

### 2.1. Patient Selection and Study Design

This retrospective cohort study included breast cancer patients, genetically tested at the Regina Maria Health Private Network, Cluj-Napoca, and treated with adjuvant radiotherapy at the “Prof. Dr. I. Chiricuță” Institute of Oncology, Cluj-Napoca, Romania. Patients were stratified by germline mutation status to evaluate the risk of acute radiotherapy-induced skin toxicity. A total of 291 female patients were included based on the following criteria: (1) histologically confirmed unilateral breast cancer diagnosed between January 2020 and December 2025; (2) absence of distant metastatic disease at the time of diagnosis (clinical stage I–III); (3) no history of other primary malignancies; (4) receipt and completion of adjuvant radiotherapy with curative intent delivered to a single breast; (5) treatment using IMRT-IP; (6) hypofractionated radiotherapy schedules consisting of 42.56 Gy in 16 fractions; (7) availability of complete radiotherapy treatment records, including dose, fractionation, and technique; (8) availability of documented genotyping results in the medical records; (9) documented assessment of acute radiation-induced skin toxicity graded from 0 to 4 according to the Common Terminology Criteria for Adverse Events (CTCAE); (10) no prior radiotherapy to the breast or thoracic region; and (11) availability of adequate clinical documentation to allow evaluation of acute radiodermatitis.

### 2.2. Confounding Variables

To control for potential confounding factors, the following lifestyle and health-related variables were included in the analysis: age, body mass index (BMI), smoking status, alcohol consumption, physical activity, comorbidities, chronic medication use, and dietary habits.

BMI was calculated as weight divided by height squared (kg/m^2^) and categorized according to World Health Organization (WHO) criteria as underweight (<18.5 kg/m^2^), normal weight (18.5–24.9 kg/m^2^), overweight (25.0–29.9 kg/m^2^), and obese (≥30.0 kg/m^2^). BMI was analyzed as a categorical variable.

Smoking status was coded dichotomously as “Yes” for participants who were current smokers or had smoked within the previous six months, and “No” for non-smokers or former smokers with abstinence > 6 months.

Alcohol consumption was similarly categorized, with “Yes” defined as intake of ≥1 unit/week (10–12 g of pure alcohol) and “No” as abstinence or intake below this threshold.

Physical activity was classified as an ordinal variable based on WHO recommendations: low (<150 min/week of moderate-intensity activity), moderate (150–300 min/week), and high (>300 min/week of moderate activity or ≥150 min/week of vigorous activity).

Comorbidities were recorded as a dichotomous variable (“Yes/No”), with “Yes” indicating the presence of at least one medically diagnosed chronic condition (e.g., hypertension, diabetes mellitus, and cardiovascular, pulmonary, or renal disease).

Chronic medication use was documented separately and coded as “Yes” for regular use of prescribed medications for chronic conditions.

Dietary habits were assessed dichotomously as “Balanced” defined by consumption of ≥5 daily servings of fruits and vegetables, regular intake of whole grains, and limited ultra-processed foods or “Unbalanced”, reflecting lower fruit and vegetable intake and frequent consumption of fast food, refined sugars, and saturated fats.

All variables were collected from patient charts and included in multivariable statistical models to adjust for potential confounding effects on the association between genetic mutation status and radiotherapy-induced skin toxicity.

Although patient-intrinsic and lifestyle variables were included as confounders, tumor histopathological characteristics (e.g., ER, PR, HER2 status, molecular subtype) were not considered. Given that all patients received adjuvant radiotherapy after surgery, the irradiated tissue consisted of normal breast/chest wall skin, so tumor-specific features are unlikely to directly affect acute radiodermatitis.

### 2.3. Genetic Analysis and Variant Interpretation

Peripheral blood samples were collected from all enrolled patients, and germline DNA was extracted using standard procedures. Genetic analyses were conducted using next-generation sequencing data generated on the Illumina platform (Illumina Inc., San Diego, CA, USA), with sequence reads aligned to the human reference genome GRCh37/hg19. Detected variants were evaluated using population and clinical reference databases, including gnomAD and ClinVar, in combination with dedicated bioinformatics tools. Variant calling was carried out with the Genome Analysis Toolkit (GATK, version 4.3.0.0; Broad Institute, Cambridge, MA, USA). Subsequent variant annotation and interpretation were performed using VarSeq (version 2.4.0; Golden Helix, Bozeman, MT, USA) and Alamut Visual (version 1.11; SOPHiA GENETICS, Rolle, Switzerland). Copy number variation analysis was conducted using ExomeDepth (version 1.1.15; University of Cambridge, Cambridge, UK). Variant pathogenicity was assessed by a multidisciplinary clinical team in accordance with ACMG criteria, and clinically relevant variants were validated by Sanger sequencing using the ProDye^®^ Terminator Sequencing System (Promega Corporation, Madison, WI, USA) and independent confirmation through Eurofins Genomics (Ebersberg, Germany).

### 2.4. Genetic Subgroup Definition and Functional Category-Based Analysis

**Figure 1:**
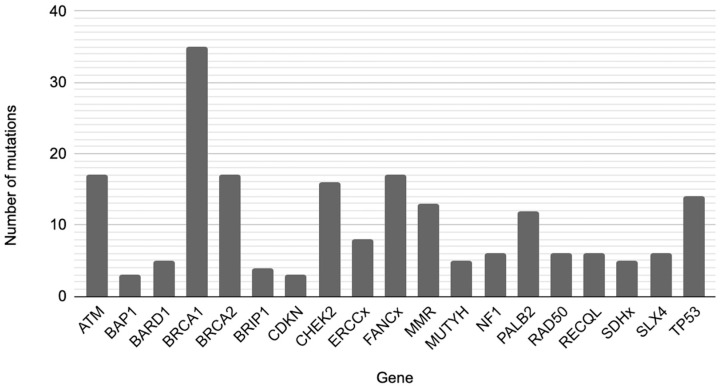
Gene-wise distribution of mutations.

For functional category-based analyses, genetic mutations were grouped according to their established functional roles in DNA damage sensing, repair, and tumor suppression. The DDR subgroup included mutations in *ATM*, *CHEK2*, and *TP53*, genes involved in damage detection, signaling, and cell cycle checkpoint activation. The Homologous Recombination Repair (HRR) subgroup comprised mutations in *BRCA1*, *BRCA2*, *PALB2*, *BARD1*, *RAD50*, *BRIP1*, and *RECQL*, which are essential for high-fidelity repair of DNA DSBs. The Fanconi Anemia (FA) pathway subgroup included mutations in core *FANC* genes as well as *BRCA1*, *BRCA2*, *PALB2*, *BRIP1*, *RAD50*, and *SLX4*, reflecting their functional cooperation in interstrand crosslink repair and genome stability maintenance. Tumor Suppressor (TS) gene analysis included mutations in *TP53*, *NF1*, and *CDKN* genes, which primarily regulate cell cycle control, genomic integrity, and cellular proliferation. The gene-wise distribution of mutations contributing to these pathway-based subgroup definitions is illustrated in Figure 1. Each functional category was analyzed independently by comparing patients harboring a mutation in the respective gene set with patients without mutations, using ordinal logistic regression models adjusted for clinical and lifestyle-related confounders.

Because nearly half of the observed mutations were in the HRR and FA pathways, we defined an additional subgroup (Non-HRR/FA) to evaluate whether the effects detected in the pooled model were independent of these dominant mutation categories. This approach ensures that the pathway-level associations are not primarily driven by the high prevalence of HRR and FA mutations.

Rather than analyzing individual point mutations, which can have variable and context-dependent effects, we focused on the molecular pathway or functional category. This approach allows multiple genes within the same pathway to collectively influence outcomes, while the effect of a single disrupted gene may be partially compensated by others. Consequently, pathway-level analysis provides a more stable and biologically meaningful assessment of the risk for radiation dermatitis compared to evaluating individual mutations.

Although the cohort could, in principle, be subdivided by additional functional categories or individual genes, the small number of patients carrying mutations within specific pathways, or mutations limited to a single gene, precluded reliable statistical analysis. Conducting separate analyses under these conditions would result in low statistical power and unstable estimates, limiting the interpretability of the findings.

### 2.5. Radiotherapy Parameters

All patients received adjuvant radiotherapy to the affected breast with curative intent using IMRT-IP which allows highly conformal dose distribution through inverse planning optimization, improving target coverage while reducing dose to surrounding organs at risk (e.g., heart and ipsilateral lung). The integrated boost technique enables simultaneous delivery of a higher dose to the tumor bed within the same treatment session, without prolonging overall treatment time, thereby maintaining biological effectiveness while optimizing dose homogeneity within the breast tissue [14].

A uniform hypofractionated schedule of 42.56 Gy delivered in 16 fractions (2.66 Gy per fraction), administered once daily, with five fractions per week, was used for all patients. Hypofractionation is supported by randomized clinical trials demonstrating equivalent tumor control and acceptable toxicity profiles compared with conventional fractionation in early-stage breast cancer. The use of a standardized hypofractionated regimen ensured consistent total dose, fraction size, and overall treatment duration across the cohort, thereby minimizing variability in radiation exposure as a potential confounding factor for acute radiodermatitis [14].

Complete radiotherapy records were available for all patients, including total dose, fractionation scheme, beam configuration, and dosimetric parameters. By restricting inclusion to patients treated with the same technique and fractionation schedule, the influence of dose and dose rate variability on acute skin toxicity was substantially reduced.

### 2.6. Applied Statistical Methods

Statistical analyses were performed using Jamovi software (version 2.6.17; The Jamovi Project, Sydney, Australia). Acute radiation-induced skin toxicity graded according to CTCAE was analyzed as an ordinal outcome. Associations between genetic mutation status and radiodermatitis severity were evaluated using ordinal logistic regression models with proportional odds assumptions. Separate models were constructed for the overall mutation status and for predefined genetic pathway subgroups. All models were adjusted for potential confounding variables. Prior to regression modeling, baseline differences between groups were assessed using independent samples *t*-tests for continuous variables and contingency table analyses with χ^2^ tests for categorical variables. Model performance was assessed using deviance, Akaike and Bayesian information criteria, and pseudo-R^2^ measures, while overall model significance was evaluated using likelihood ratio χ^2^ tests. Results are reported as regression coefficients, odds ratios (OR) with 95% confidence intervals (95% CI), and corresponding *p*-values. A two-sided *p*-value < 0.05 was considered statistically significant.

## 3. Results

The results of the descriptive and inferential statistical analyses are summarized in Table 1, Table 2, Table 3 and Table 4. These tables present baseline group comparisons and the findings of the ordinal logistic regression models evaluating the association between genetic mutation status and the severity of acute radiation-induced skin toxicity.

Table 1 presents the baseline characteristics of the study cohort (*n* = 291), stratified by genetic mutation status (negative vs. positive for pathogenic variants) and by specific mutation subgroups, including DDR, HRR, FA, TS, and Non-HRR/FA. Continuous variables, such as age, were compared between groups using independent samples *t*-tests, while categorical variables, including BMI, smoking status, alcohol consumption, physical activity, comorbidities, chronic medication use, and diet, were analyzed using χ^2^ tests for independence. This analysis aimed to identify potential differences in demographic and lifestyle characteristics across mutation status and subgroups, which could act as confounding variables in subsequent regression modeling of genetic predictors of acute radiodermatitis.

**Table 1:** Comparison of baseline characteristics across genetic mutation groups.

Confounding Variable	Study Cohort (*n* ^1^ = 291)	Negative (*n* = 93)	Positive (*n* = 198)	*p* ^2^	DDR (*n* = 47)	*p*	HRR (*n* = 85)	*p*	FA (*n* = 97)	*p*	TS (*n* = 23)	*p*	Non-HRR/FA (*n* = 95)	*p*
Age ^3^	46.30 ± 9.58	45.20 ± 8.91	46.80 ± 9.86	0.180	46.70 ± 9.95	0.345	44.80 ± 9.30	0.804	45.60 ± 9.52	0.733	50.10 ± 12.46	0.031	47.70 ± 10.18	0.072
BMI														
Normal weight	133	44	89	0.572	18	0.582	40	0.637	42	0.600	10	0.873	44	0.764
Overweight	104	35	69		20		28		35		10		33	
Obese	54	14	40		9		17		20		3		18	
Smoking														
No	207	66	141	0.966	31	0.544	65	0.405	65	0.556	19	0.259	71	0.561
Yes	84	27	57		16		20		32		4		24	
Alcohol														
No	238	80	158	0.200	38	0.427	69	0.382	75	0.122	18	0.357	77	0.359
Yes	53	13	40		9		16		22		5		18	
Physical activity														
Low	107	33	74	0.523	18	0.737	35	0.264	40	0.425	7	0.704	32	0.638
Moderate	138	48	90		25		34		41		14		46	
High	46	12	34		4		16		16		2		17	
Comorbidities														
No	182	61	121	0.462	29	0.650	55	0.901	58	0.409	12	0.233	58	0.519
Yes	109	32	77		18		30		39		11		37	
Chronic Medication														
No	211	73	138	0.117	35	0.592	66	0.891	68	0.186	14	0.080	65	0.118
Yes	80	20	60		12		19		29		9		30	
Diet														
Balanced	143	45	98	0.860	23	0.951	40	0.859	47	0.993	12	0.745	47	0.882
Unbalanced	148	48	100		24		45		50		11		48	

Note: ^1^ *n*, number of patients. ^2^ *p*-values were derived from independent samples *t*-tests for the continuous variable (age) and from χ^2^ tests for categorical variables (BMI, smoking status, alcohol consumption, physical activity, comorbidities, chronic medication use, and diet). ^3^ Age values are expressed as mean ± standard deviation.

Overall, baseline demographic, clinical, and lifestyle characteristics were largely comparable between patients without genetic mutations and those harboring mutations, including the DDR, HRR, FA, TS, and Non-HRR/FA subgroups (Table 1). Age did not differ significantly between the negative and positive genetic mutation groups overall, nor across most functional gene categories; however, patients in the TS subgroup were significantly older compared with their respective reference group (*p* = 0.031). No statistically significant differences were observed for BMI, smoking status, alcohol consumption, physical activity level, comorbidities, chronic medication use, or dietary pattern across mutation statuses (all *p* > 0.05; Table 1). This relative balance of potential confounding variables supports the robustness of subsequent ordinal logistic regression analyses and suggests that observed associations between genetic predictors and acute radiodermatitis are unlikely to be driven by systematic differences in baseline characteristics.

Table 2 summarizes the goodness-of-fit indices and overall model performance for the ordinal logistic regression analyses evaluating the association between genetic mutation status and acute radiotherapy-induced skin toxicity. Separate ordinal logistic regression models were constructed for the pooled mutation analysis (positive vs. negative genetic mutation status) and for each functional gene category (DDR, HRR, FA, TS, Non-HRR/FA) compared to the negative group. Model adequacy was assessed using deviance, Akaike Information Criterion (AIC), Bayesian Information Criterion (BIC), and multiple pseudo-R^2^ measures (McFadden’s, Cox & Snell’s, and Nagelkerke’s). Overall model significance was evaluated using likelihood ratio chi-square tests.

**Table 2:** Ordinal Logistic Regression Model Fit for Genetic Predictors of Acute Radiodermatitis.

Model	Deviance	AIC ^1^	BIC ^2^	R^2^_McF_ ^3^	R^2^_CS_ ^4^	R^2^_N_ ^5^	Overall Model Test	
							χ^2^	*p*
Pool	573	601	653	0.0382	0.0155	0.0462	22.8	0.012
DDR	269	297	338	0.0577	0.0232	0.0695	16.5	0.087
HRR	358	386	431	0.0583	0.0246	0.0708	22.2	0.014
FA	377	405	450	0.0572	0.0238	0.0692	22.9	0.011
TS	211	239	277	0.0676	0.0260	0.0805	15.3	0.123
Non-HRR/FA	353	381	426	0.0486	0.0190	0.0583	18.0	0.054

Note: ^1^ AIC = Akaike Information Criterion. ^2^ BIC = Bayesian Information Criterion. ^3^ R^2^_McF_ = McFadden’s R^2^. ^4^ R^2^_CS_ = Cox & Snell’s R^2^. ^5^ R^2^_N_ = Nagelkerke’s R^2^.

Table 3 presents the omnibus likelihood ratio test results for each ordinal logistic regression model, assessing whether the inclusion of genetic mutation status significantly improved model fit compared with the intercept-only model. These tests provide a global assessment of the contribution of mutation status to the prediction of radiodermatitis severity across the ordered CTCAE toxicity grades.

**Table 3:** Omnibus Model Tests for Genetic Mutation Status and Acute Skin Toxicity.

Model	χ^2^	*p*
Pool	9.052	0.003
DDR	3.761	0.052
HRR	9.594	0.002
FA	10.010	0.002
TS	0.766	0.381
Non-HRR/FA	3.486	0.062

The performance of the ordinal logistic regression models varied according to the genetic grouping strategy, highlighting the value of biologically informed, functional category-based analyses. As shown in Table 2, the pooled mutation model (positive vs. negative genetic mutation status) demonstrated statistically significant overall model fit (overall model test: χ^2^ = 22.8, *p* = 0.012), indicating that the inclusion of mutation status significantly improved the prediction of acute radiodermatitis severity compared with an intercept-only model. However, the pseudo-R^2^ values for this model were modest (McFadden’s R^2^ = 0.0382; Nagelkerke’s R^2^ = 0.0462), reflecting the limited proportion of outcome variability explained by genetic status alone. This magnitude is consistent with clinical toxicity models, in which outcomes are influenced by multiple interacting biological and treatment-related factors.

In addition to the overall model test reported in Table 2, an omnibus likelihood ratio test was performed for each model (Table 3). While both tests rely on likelihood-based comparisons, they are reported separately because they address related but distinct aspects of model evaluation. The overall model test in Table 2 reflects the improvement in model fit after inclusion of all predictors relative to the null model, whereas the omnibus likelihood ratio test in Table 3 specifically assesses the contribution of mutation status to explaining variability in radiodermatitis grade. In the pooled model, both tests were statistically significant (Table 2: χ^2^ = 22.8, *p* = 0.012; Table 3: χ^2^ = 9.052, *p* = 0.003), providing convergent evidence that mutation status is a relevant predictor of acute skin toxicity severity.

By contrast, when the pooled analysis excluded mutations in the HRR and FA pathways (Non-HRR/FA), the model lost statistical significance (overall model test: χ^2^ = 18.0, *p* = 0.054; McFadden’s R^2^ = 0.0486; Nagelkerke’s R^2^ = 0.0583; omnibus likelihood ratio test: χ^2^ = 3.486, *p* = 0.064). This loss of significance is likely since nearly half of all mutations in the cohort are found in the HRR and FA pathways. Therefore, the predictive signal observed in the full pooled model appears to be largely driven by these two abundant mutation groups, suggesting that the pathway-level associations detected in the overall pooled analysis are, at least in part, artifacts of mutation preponderance rather than reflecting contributions from other, less frequent mutations. These results further support the importance of analyzing functional categories with sufficient representation in the cohort to obtain biologically meaningful and statistically reliable estimates.

Functional category-specific analyses revealed superior and more consistent performance for the HRR and FA models, both of which demonstrated statistically significant overall model tests (Table 2: HRR χ^2^ = 22.2, *p* = 0.014; FA χ^2^ = 22.9, *p* = 0.011) and significant omnibus likelihood ratio tests (Table 3: HRR χ^2^ = 9.594, *p* = 0.002; FA χ^2^ = 10.01, *p* = 0.002). These models also showed higher pseudo-R^2^ values compared with the pooled analysis (Nagelkerke’s R^2^ = 0.0708 for HRR and 0.0692 for FA), indicating improved explanatory power when mutations were grouped according to functionally coherent DNA repair mechanisms.

The similarity in model performance metrics between HRR and FA is biologically expected and reflects their partial gene overlap and functional interdependence. Several genes (*BRCA1*, *BRCA2*, *PALB2*, *BRIP1*, *RAD50*) contribute to both homologous recombination and Fanconi-mediated interstrand crosslink repair. As a result, these models capture overlapping dimensions of DNA repair deficiency, leading to comparable χ^2^ statistics, pseudo-R^2^ values, and model fit indices. Importantly, this convergence does not indicate redundancy but rather reinforces the central role of high-fidelity DNA repair capacity in determining normal tissue radiosensitivity.

In contrast, the DDR and TS models did not reach statistical significance at the global level (Table 2; DDR: χ^2^ = 16.5, *p* = 0.087; TS: χ^2^ = 15.3, *p* = 0.123), nor did they demonstrate significant omnibus likelihood ratio tests (Table 3). Although the TS model exhibited the highest Nagelkerke’s R^2^ (0.0805), this apparent explanatory capacity did not translate into statistically significant improvement over the null model, likely reflecting increased variability and reduced precision due to functional heterogeneity within the gene group. Collectively, these findings indicate that predictive strength is maximized when genetic alterations are grouped according to functional categories directly involved in DNA damage repair, rather than broader signaling or tumor suppressor functions.

Table 4 details the regression coefficients, standard errors, Wald statistics, *p*-values, and corresponding ORs with 95% CI for genetic mutation status across all models. ORs represent the relative odds of experiencing a higher grade of acute radiation-induced skin toxicity associated with the presence of a mutation compared with no detected mutation, after accounting for the ordinal nature of the outcome.

**Table 4:** Effect Estimates of Genetic Mutations on the Severity of Radiation-Induced Skin Toxicity.

Model	β ^1^	95% CI		SE ^2^	Z ^3^	*p*	OR	95% CI	
		Lower	Upper					Lower	Upper
Pool	0.807	0.2768	1.3593	0.275	2.929	0.003	2.240	1.319	3.893
DDR	0.729	−0.0078	1.472	0.376	1.937	0.053	2.072	0.992	4.359
HRR	0.957	0.348	1.5824	0.314	3.046	0.002	2.603	1.417	4.866
FA	0.963	0.363	1.583	0.311	3.100	0.002	2.619	1.437	4.870
TS	0.426	−0.539	1.370	0.484	0.881	0.378	1.532	0.584	3.934
Non-HRR/FA	0.579	−0.0286	1.202	0.313	1.850	0.064	1.785	0.972	3.328

Note: ^1^ Estimate (β), the regression coefficient; ^2^ SE, standard error of the estimate; ^3^ Z, the z-statistic calculated as Estimate divided by SE, indicating the significance of the effect.

The regression coefficients and effect estimates (β) presented in Table 4 provide detailed insight into the magnitude and direction of the associations between genetic mutation status and acute radiodermatitis severity. In the pooled mutation analysis, the presence of a mutation was associated with a statistically significant increase in toxicity severity (Table 4; β = 0.807, SE = 0.275, Z = 2.929, *p* = 0.003), corresponding to an odds ratio of 2.24 (95% CI: 1.32–3.89). This indicates that patients with documented mutations had more than double the odds of experiencing a higher grade of acute radiodermatitis compared with patients without mutations, after adjustment for relevant confounders. The significance of this association is corroborated by the omnibus likelihood ratio test (Table 3, χ^2^ = 9.052, *p* = 0.003), confirming the independent contribution of mutation status to toxicity risk.

By contrast, the pooled analysis excluding HRR and FA mutations (Non-HRR/FA) showed no significant association (β = 0.579, SE = 0.313, Z = 1.850, *p* = 0.064; OR = 1.785, 95% CI: 0.972–3.328; omnibus test χ^2^ = 3.486, *p* = 0.064), indicating that the effect observed in the full pooled model is largely driven by these abundant mutation groups.

Functional category-specific analyses revealed that this increased risk was primarily driven by mutations in DNA repair-related functional categories directly involved in the resolution of radiation-induced DNA damage. HRR mutations were associated with a significant increase in radiodermatitis severity (Table 4; β = 0.957, *p* = 0.002, OR = 2.60, 95% CI: 1.42–4.87). Similarly, FA mutations demonstrated a comparable and statistically significant effect (Table 4; β = 0.963, *p* = 0.002, OR = 2.62, 95% CI: 1.44–4.87). The close alignment of effect sizes, confidence intervals, and *p*-values between these two pathways reflects their shared molecular architecture and overlapping gene composition, as well as their coordinated role in repairing complex DNA lesions induced by ionizing radiation.

The partial overlap between HRR and FA genes likely amplifies the observed associations, as dysfunction in shared components such as BRCA1/2 and PALB2 simultaneously compromises double-strand break repair and interstrand crosslink resolution. This compounded repair deficiency may exacerbate keratinocyte damage, delay epidermal regeneration, and intensify inflammatory responses, thereby increasing susceptibility to higher-grade acute radiodermatitis. The statistical consistency across HRR and FA models, supported by significant omnibus tests (Table 3), strengthens the biological plausibility of these findings.

In contrast, DDR mutations showed only a borderline association with toxicity severity (Table 4; β = 0.729, *p* = 0.053; OR = 2.07, 95% CI: 0.99–4.36), mirrored by a non-significant omnibus test (Table 3; χ^2^ = 3.76, *p* = 0.052). This suggests that genes primarily involved in damage sensing and checkpoint signaling may influence radiosensitivity indirectly or variably, resulting in less consistent effects on acute skin toxicity. TS gene mutations showed no significant association with radiodermatitis severity (Table 4; β = 0.426, *p* = 0.378; OR = 1.53, 95% CI: 0.58–3.93), indicating that alterations affecting cell cycle control or oncogenic suppression do not substantially modulate acute cutaneous radiation response in this cohort.

Taken together, these results demonstrate that the increased risk of acute radiotherapy-induced skin toxicity is selectively associated with mutations in core DNA repair pathways, particularly those with overlapping roles in homologous recombination and Fanconi-mediated repair. The consistency of statistical significance across multiple model evaluation metrics and effect estimates highlights the robustness of this association and supports the biological relevance of functional category-based genetic stratification in predicting radiodermatitis severity.

## 4. Discussion

### 4.1. Biological Interpretation of DDR Functional Category Results

The lack of a statistically significant association between DDR functional category mutations and acute radiodermatitis severity observed in our cohort is biologically plausible and consistent with current molecular evidence regarding the functional role of DDR signaling in cellular radioresponse. DDR genes are primarily responsible for damage sensing, signal transduction, and cell cycle checkpoint activation, rather than direct DNA lesion repair. Core DDR components such as ATM, ATR, DNA-PKcs, CHEK2, and TP53 orchestrate complex signaling cascades that promote cell cycle arrest and facilitate the activation of downstream repair mechanisms, including non-homologous end joining (NHEJ) and HRR [7].

ATM, as a central DDR kinase, plays a pivotal role in coordinating checkpoint activation and repair pathway selection following ionizing radiation-induced DSBs. Upon activation, ATM triggers the ATM–CHK2 and ATR–CHK1 signaling cascades, inducing G1/S, S, and G2/M cell cycle arrest, thereby providing cells with sufficient time to repair DNA damage [7,15,16,17]. Importantly, multiple studies have demonstrated that increased ATM activity and intact DDR checkpoint signaling are associated with radioresistance, as prolonged checkpoint arrest enhances DNA repair efficiency and promotes cellular survival after irradiation [7,17]. Conversely, suppression or pharmacologic inhibition of ATM disrupts checkpoint control and increases radiosensitivity, a principle currently exploited therapeutically through DDR-targeted inhibitors [17].

This biological framework provides a mechanistic explanation for the weak and borderline association observed in our DDR model. Mutations affecting DDR signaling genes (*ATM*, *CHEK2*, *TP53*) do not uniformly result in increased radiosensitivity. Instead, their functional consequences are heterogeneous, ranging from impaired checkpoint control to compensatory activation of alternative pathways such as ATR–CHK1–WEE1 signaling, which can maintain cell cycle arrest and DNA repair capacity despite upstream defects [17]. Tumor and normal cells with defective *ATM* or *TP53* frequently develop increased reliance on S/G2 checkpoint mechanisms, thereby preserving genomic stability and limiting radiosensitivity through compensatory pathway rewiring [17].

Furthermore, DDR alterations have been repeatedly associated with radioresistance rather than radiosensitivity in multiple malignancies. High ATM expression levels correlate with resistance to radiotherapy, and intact ATM–CHK2 signaling promotes efficient repair, restoration of cell cycle progression, and cellular survival after radiation exposure [7]. ATM-mediated regulation of p53 degradation and checkpoint recovery further contributes to radioresistant phenotypes by allowing damaged cells to resume proliferation following repair [7]. These mechanisms suggest that DDR mutations may not consistently increase radiation-induced tissue toxicity but may instead produce variable or even protective effects depending on the specific mutation, zygosity, and compensatory signaling context.

Network-based analyses further support this interpretation. Interaction studies using STRING and GeneMania demonstrate that *ATM*, *ATR*, *CHEK1*, *CHEK2*, and *TP53* participate in extensive and highly interconnected signaling networks, including homologous recombination, FA, p53 signaling, and mismatch repair pathways [15]. This extensive crosstalk implies that isolated DDR gene mutations may be functionally buffered by parallel repair and checkpoint pathways, thereby diluting their clinical impact on normal tissue radiosensitivity.

Clinically, germline and somatic mutations in *ATM*, *CHEK2*, and *TP53* are strongly associated with tumorigenesis, genomic instability, and therapy resistance, rather than increased normal tissue toxicity [16,17]. While homozygous *ATM* mutations cause profound radiosensitivity syndromes such as ataxia-telangiectasia, heterozygous pathogenic variants—more representative of clinical cancer cohorts—are primarily linked to cancer predisposition and clonal expansion, not to exaggerated normal tissue radiation responses [16]. Similarly, *CHEK2* and *TP53* mutations are associated with defective checkpoint control and malignant transformation but do not consistently translate into heightened radiosensitivity of normal tissues [16,17].

Together, these molecular and clinical data align closely with our statistical findings. The borderline and non-significant association between DDR functional category mutations and acute radiodermatitis severity reflects the biological role of DDR genes as regulatory and signaling modulators, rather than direct executors of DNA repair. In contrast, functional categories directly responsible for high-fidelity DNA lesion repair, such as HRR and FA category, show strong and consistent associations with toxicity risk in our cohort. This supports the concept that defects in core repair machinery, rather than in upstream damage sensing and checkpoint signaling, are the principal determinants of normal tissue radiosensitivity and reinforce the value of functional genetic categorization for biologically informed risk stratification.

### 4.2. Biological Interpretation of HRR Pathway Results

From a clinical perspective, identification of HRR and FA mutations as predictors of acute skin toxicity may support pre-treatment risk stratification. Patients harboring such alterations could benefit from intensified skin care protocols, closer toxicity monitoring, or adaptive planning strategies to minimize cutaneous dose. While genetic testing is not yet routinely implemented for toxicity prediction, functional category-based models offer a scalable framework for integrating germline data into personalized radiotherapy planning.

The superior performance of the HRR pathway model in our analysis provides a coherent statistical–biological framework for understanding individual susceptibility to acute radiotherapy-induced skin toxicity. Among all genetic grouping strategies evaluated, HRR mutations demonstrated both statistically significant global model fit and a robust independent association with radiodermatitis severity, as reflected by the overall model test (χ^2^ = 22.2, *p* = 0.014), the omnibus likelihood ratio test (χ^2^ = 9.594, *p* = 0.002), and an adjusted OR of 2.60 for higher toxicity grades. The corresponding increase in pseudo-R^2^ values compared with the pooled mutation model indicates that grouping mutations according to homologous recombination function improves explanatory power and model coherence.

The strong association between HRR pathway mutations and increased severity of acute radiotherapy-induced skin toxicity observed in our cohort is supported by the central molecular functions of the HRR machinery in maintaining genome stability after ionizing radiation exposure. HRR is responsible for the accurate repair of DSBs using an intact sister chromatid as a template, thereby preventing error-prone repair, and preserving cellular viability. Disruption of this pathway results in accumulation of unresolved DSBs, chromosomal aberrations, and increased cellular radiosensitivity [7].

BRCA1 and BRCA2 represent core scaffolding proteins within the HRR pathway, coordinating end resection, RAD51 filament formation, and strand invasion. Loss-of-function alterations in BRCA1/2 impair RAD51 recruitment and strand exchange, leading to defective DSB repair and enhanced sensitivity to radiation-induced DNA damage. PALB2 serves as a critical molecular bridge between BRCA1 and BRCA2, stabilizing the HRR complex and facilitating efficient RAD51 loading at sites of damage. Mutations in *PALB2* therefore phenocopy BRCA1/2 deficiency by disrupting the structural integrity of the HRR axis [7,18,19].

BARD1 functions as an obligate binding partner of BRCA1, forming a heterodimer with E3 ubiquitin ligase activity that regulates DNA end resection and chromatin remodeling at DSB sites. Pathogenic variants in *BARD1* destabilize *BRCA1* function and further compromise homologous recombination efficiency. RAD50, as a component of the MRN complex (MRE11–RAD50–NBS1), plays a key role in DSB detection, tethering of DNA ends, and initiation of HRR. Defects in RAD50 impair DSB processing and limit the activation of downstream homologous recombination machinery, thereby increasing vulnerability to radiation-induced genomic injury [18,20].

BRIP1 (also known as FANCJ) is a DNA helicase that interacts directly with BRCA1 and participates in DNA end processing and replication fork stability. Mutations in *BRIP1* reduce helicase activity and impair HRR-mediated repair, contributing to replication stress and increased radiosensitivity. RECQL, a member of the RecQ helicase family, is involved in maintaining replication fork integrity and facilitating HRR-mediated repair of stalled or collapsed replication forks. Loss of *RECQL* function leads to replication-associated DNA damage accumulation and defective homologous recombination, further sensitizing cells to ionizing radiation [21,22].

In normal tissues such as the skin, where basal keratinocytes undergo continuous proliferation, coordinated HRR activity is essential to preserve genomic integrity following radiotherapy. Impairment of any of these HRR components compromises the ability of epidermal cells to resolve radiation induced DSBs efficiently, resulting in increased cell death, delayed re-epithelialization, and enhanced inflammatory responses. These molecular mechanisms provide a plausible explanation for the statistically robust association observed in our ordinal regression models, where HRR mutations were associated with a more than twofold increase in the odds of higher-grade acute radiodermatitis [18,23].

Importantly, the improved performance of the HRR model compared with broader genetic groupings reflects the direct involvement of these genes in high-fidelity DNA repair rather than upstream signaling or checkpoint control. The convergence of statistical robustness and molecular function supports the interpretation that alterations in core HRR components constitute a biologically coherent and clinically informative determinant of acute normal tissue radiosensitivity, with potential utility for risk stratification in the radiotherapy setting.

### 4.3. Biological Interpretation of FA Pathway Results

The strong and statistically consistent association observed for FA alterations, reflected by significant overall model fit, omnibus likelihood ratio testing, and an OR exceeding 2.60 for higher-grade acute radiodermatitis, highlights the central role of replication-associated DNA repair in normal tissue radiosensitivity. Unlike broader genetic groupings, the FA model captures a functionally coherent DNA repair program specifically dedicated to the resolution of replication stress, DNA interstrand crosslinks (ICLs), and radiation-induced replication fork instability, which are critical determinants of epidermal cell survival following ionizing radiation.

To date, mutations in at least 22 *FANC* genes (*FANCA*–*FANCW*) have been identified, encoding proteins that assemble into a multi-module repair pathway orchestrating ICL repair, replication fork protection, and replication restart [16,24,25]. Several mechanistic models have been proposed to explain FA-mediated ICL repair, including the single-fork collapse model, the converging double-fork model, and the ICL traverse model, the latter accounting for up to 50–60% of replication events and critically dependent on FANCM translocase activity [16]. Despite mechanistic differences, all models converge on the generation of one-ended or double-ended DSBs, which must ultimately be resolved through homologous recombination or break-induced replication, positioning FA as a replication-coupled extension of the HRR machinery [12,16].

Loss-of-function alterations in FA components result in persistent replication stress, stalled, or collapsed replication forks, and accumulation of unrepaired DSBs, leading to constitutive activation of ATM- and ATR-dependent DNA damage response signaling [16,24]. This chronic DDR activation drives sustained engagement of the p53–p21 axis, cell cycle arrest, senescence, and apoptosis, all of which are hallmarks of Fanconi-deficient cells [16,25]. In highly proliferative tissues such as the epidermis, these processes are expected to impair keratinocyte renewal, delay re-epithelialization, and exacerbate inflammatory signaling following radiation exposure, thereby increasing susceptibility to higher-grade acute radiodermatitis [16]. The magnitude and statistical robustness of the FA-associated effect observed in this study are therefore biologically congruent with the established cellular phenotype of Fanconi pathway deficiency.

Importantly, the predictive strength of the FA model mirrors that of the HRR model, a finding that is biologically expected given their shared molecular architecture. Several FA genes, including *FANCD1/BRCA2*, *FANCN/PALB2*, *FANCJ/BRIP1*, *FANCS/BRCA1* and *FANCO/RAD51C*, directly encode HR-associated proteins, and germline monoallelic mutations in these genes predispose to familial breast and ovarian cancer syndromes [12]. This partial gene overlap likely amplifies the observed association with radiodermatitis severity by simultaneously compromising replication fork rescue and high-fidelity DSB repair, thereby intensifying radiation-induced tissue damage [7,16].

Although the global DDR model did not reach statistical significance in this cohort, substantial functional overlap exists between the FA and core DDR signaling networks, providing a mechanistic basis for the borderline effects observed for DDR-associated genes. The *FANC/BRCA* pathway is embedded within the broader DDR architecture and is tightly regulated by ATM and ATR kinases, which phosphorylate multiple FANC proteins, including FANCD2, FANCI, FANCM and the E3 ligase RFWD3/FANCW, in response to replication stress and ionizing radiation [24,25]. Notably, ATR-dependent activation of the FA pathway follows a partially non-canonical mechanism that does not strictly require the RAD9–RAD1–HUS1 (9-1-1) complex or TopBP1, distinguishing FA-mediated DDR engagement from classical checkpoint signaling [24].

This intimate but specialized relationship may explain why DDR gene grouping alone lacked sufficient predictive precision: while DDR sensors and checkpoint mediators modulate damage signaling and cell cycle arrest, FA alterations directly impair the execution of DNA repair and replication recovery. As a result, mutations in FA genes exert a more consistent and biologically proximal effect on radiation-induced tissue injury than alterations confined to upstream damage sensing. The selective statistical significance of the FA model thus supports the conclusion that defects in replication-coupled DNA repair, rather than generalized DDR activation, are key drivers of acute radiodermatitis risk.

### 4.4. Biological Interpretation of TS Functional Category Results

TS genes, including *TP53*, *NF1*, and *CDKN2A/CDKN2B*, serve as central guardians of genomic stability, coordinating DDR, cell cycle control, senescence, and apoptosis [26,27,28,29]. *TP53* encodes the transcription factor p53, which is activated in response to DNA damage, oxidative stress, or oncogenic signaling. Activated p53 induces transcription of downstream effectors such as p21, GADD45, and BAX, orchestrating cell cycle arrest, DNA repair, or apoptosis depending on the severity of damage [26]. *TP53* function is tightly regulated by negative feedback through MDM2, which ubiquitinates p53 for proteasomal degradation, and is modulated by upstream signaling pathways, including STAT3, Raf/MEK/ERK, and NF-κB, linking oncogenic stress to cell fate decisions [26]. In epidermal keratinocytes, intact p53 is critical for coordinating repair and eliminating severely damaged cells, but partial loss-of-function or heterozygous mutations may be compensated by redundant DDR and checkpoint mechanisms [26,27].

*NF1* encodes neurofibromin, a GTPase-activating protein that negatively regulates Ras signaling and multiple downstream cascades, including Raf/MEK/ERK, PI3K/AKT/mTOR, and Ral/NF-κB pathways [27]. Loss-of-function *NF1* mutations result in constitutive Ras-GTP accumulation, promoting hyperactivation of proliferation and survival pathways. While these changes can increase cellular stress and indirectly influence DNA damage responses, NF1 deficiency does not directly impair homologous recombination or base excision repair mechanisms, explaining the weak association with acute radiation-induced skin toxicity observed in our cohort [26,27].

*CDKN2A* encodes two tumor suppressor proteins, p16^Ink4a^ and p14^ARF^, which independently regulate cell cycle checkpoints [28]. p16^Ink4a^ inhibits CDK4/6-mediated phosphorylation of the retinoblastoma protein, maintaining G1 arrest, whereas p14^ARF^ stabilizes p53 by sequestering MDM2, thereby reinforcing the p53-dependent DDR. Loss-of-function mutations or homozygous deletions of *CDKN2A* release G1/S and G2/M checkpoints, increasing proliferation and genomic instability [28,29]. However, the pathway primarily modulates cell cycle control rather than executing direct DNA repair, limiting its influence on acute radiotherapy-induced epidermal injury.

In our study, the TS model did not reach statistical significance, and TS mutations were not associated with higher-grade acute radiodermatitis. These findings are consistent with the molecular biology of TS genes: while they regulate proliferation, apoptosis, and senescence, they do not directly participate in the resolution of radiation-induced DSBs or replication stress, the primary drivers of keratinocyte damage and acute skin toxicity. Moreover, functional redundancy in checkpoint signaling, compensation by intact DNA repair pathways, and the multicellular architecture of the epidermis likely buffer the impact of single TS gene mutations on acute radiation response.

Collectively, these results highlight that pathway-specific functional relevance is critical: HRR and FA mutations, which directly impair high-fidelity DNA repair, confer the most consistent risk for acute radiodermatitis, whereas TS gene alterations, despite their central role in tumor suppression, are insufficient alone to modulate acute skin toxicity. These findings establish functional category-based germline variant stratification as a molecularly informed, predictive diagnostic approach for radiosensitivity assessment, and reinforce the value of biologically informed risk stratification frameworks in clinical contexts. Importantly, the data emphasize that not all genomic alterations with oncogenic or tumor suppressive relevance function as clinically informative biomarkers for normal tissue toxicity, underscoring the need for pathway-specific diagnostic evaluation.

### 4.5. Role of Confounding Factors

Radiotherapy-induced skin toxicity is a multifactorial outcome influenced by a complex interaction between treatment-related, biological, and patient-specific factors. Variables such as age, body mass index, smoking status, alcohol consumption, physical activity, comorbidities, chronic medication use, and dietary habits have all been previously implicated in modifying normal tissue response to radiation [30]. In the present study, these factors were included as covariates in all ordinal logistic regression models to mitigate their potential confounding effects on the association between genetic mutation status and radiodermatitis severity. The persistence of statistically significant associations for the pooled mutation analysis, as well as for the HRR and FA functional category models, after adjustment for these variables suggests that the observed effects are not solely attributable to differences in baseline clinical or lifestyle characteristics.

Importantly, adjustment for confounders likely contributed to the attenuation of associations observed for the DDR and TS functional category models, which did not reach statistical significance. This may indicate that the influence of mutations within these functional categories is more susceptible to modification by non-genetic factors, or that their contribution to acute skin toxicity operates through indirect mechanisms that are overshadowed by clinical determinants such as tissue perfusion, inflammatory status, or metabolic comorbidity burden. By explicitly accounting for these confounders, the analysis enhances the specificity of the observed associations and supports the interpretation that defects in core DNA repair functional categories—particularly those involving the HRR pathway and FA functional category—represent independent and biologically meaningful risk factors for radiotherapy-induced skin toxicity. Nonetheless, residual confounding cannot be entirely excluded, and future prospective studies incorporating more granular treatment-related and molecular covariates may further refine risk stratification models.

### 4.6. Limitations and Future Directions

This study has several limitations that should be considered when interpreting its findings. First, the retrospective design precludes causal inference and may be subject to selection bias. However, the use of a homogeneous treatment cohort, standardized hypofractionated IMRT protocols, and consistent CTCAE-based toxicity assessment strengthens internal validity.

Another important limitation relates to the distribution of genetic alterations within our cohort. Nearly half of the observed mutations were concentrated in the HRR and FA pathways, which likely drove much of the signal detected in the pooled mutation analyses. Consequently, pathway-level associations may disproportionately reflect the contribution of these highly represented mutation groups, whereas the effects of less frequent mutations remain difficult to evaluate. Future studies with larger cohorts are therefore required to ensure adequate representation across all functional categories, enabling more balanced pathway-level analyses and robust assessment of rare variants.

In addition, almost all germline mutations were heterozygous, with only a small proportion of homozygous variants (13 of 198). This limited distribution precluded separate statistical analyses according to zygosity. Future investigations correlating mutation type and zygosity with clinical outcomes may provide important mechanistic insights into how these genes influence normal tissue radiosensitivity and should be considered a priority for subsequent research.

The composition of the study population represents another constraint. The cohort consisted exclusively of female breast cancer patients, limiting the direct extrapolation of these findings to male breast cancer cases. Sex-related biological differences—including hormonal milieu and skin characteristics—as well as lifestyle and behavioral factors incorporated as confounders in our analysis, may influence radiation-induced skin toxicity profiles.

Despite adjustment for a comprehensive set of clinical and lifestyle-related confounders, residual confounding from unmeasured variables—such as detailed skin dose–volume parameters, baseline skin characteristics, or systemic inflammatory status—cannot be fully excluded.

Furthermore, the results are specific to adjuvant hypofractionated IMRT-IP in breast cancer and should not be generalized to other malignancies without caution. Radiotherapy protocols vary substantially across tumor types with respect to anatomical site, irradiated volume, tissue composition, dose distribution, treatment intent (neoadjuvant versus adjuvant), fractionation schedules, and tumor depth. Each of these factors may independently influence normal tissue toxicity risk and interact with underlying genetic susceptibility.

Beyond cohort- and treatment-specific considerations, the modest effect sizes and pseudo-R^2^ values observed across models indicate that germline genetic information alone provides limited predictive power. This finding underscores the multifactorial nature of normal tissue radiosensitivity and highlights the need for integrative diagnostic models that incorporate genetic, clinical, and treatment-related factors.

A further methodological consideration relates to the functional overlap between genetic categories, particularly between HRR and FA. While this overlap reflects established biological interdependence and was intentionally incorporated to capture coordinated DNA repair processes, it introduces complexity when attempting to isolate category-specific effects and may partially explain similarities in model performance and effect estimates. Conversely, the absence of robust associations for DDR and TS categories suggests that alterations in damage sensing, checkpoint control, or tumor suppressor functions may exert more indirect or context-dependent effects on acute skin toxicity. Such effects may not be fully detectable within the current analytical framework or through category-based stratification alone.

From a translational perspective, the consistent and biologically plausible associations observed for HRR and FA category mutations support their potential role as predictive molecular biomarkers of increased normal tissue radiosensitivity. Nevertheless, clinical implementation requires careful validation. Before integration into routine radiotherapy decision-making or pre-treatment screening strategies, prospective validation in larger, multicenter cohorts with diverse patient populations is essential to confirm reproducibility and generalizability.

Future research should integrate functional category-based genomic profiling with high-resolution dosimetric data, longitudinal toxicity phenotyping, and complementary molecular biomarkers to enhance predictive accuracy and clinical relevance. In parallel, functional and mechanistic studies are warranted to clarify how alterations in DDR, HRR, FA, and TS pathways influence epidermal DNA repair capacity, inflammatory signaling, and tissue regeneration following radiation exposure. Ultimately, the development and validation of multifactorial predictive models that combine genetic, clinical, and treatment-related variables may yield clinically actionable decision-support tools for personalized radiotherapy—aimed at minimizing toxicity while preserving oncologic efficacy and advancing precision radiation oncology.

## 5. Conclusions

This study shows that germline mutations in DNA repair functional categories, particularly HRR and FA-associated genes, are linked to increased severity of acute radiodermatitis in breast cancer patients treated with hypofractionated IMRT-IP. In pooled analyses, the presence of any mutation increased the odds of higher-grade toxicity (OR = 2.24); however, when HRR and FA mutations were excluded from the pooled analysis, the association was no longer significant (*p* = 0.064). Mutations specifically in HRR (OR = 2.60) and FA (OR = 2.62) substantially increased the risk of severe toxicity. These findings underscore the critical role of high-fidelity DNA repair in epidermal radiosensitivity, with overlapping defects in HRR and FA genes amplifying keratinocyte damage and delaying tissue recovery. By contrast, mutations in DDR or TS genes showed non-significant associations, consistent with their more indirect contribution to DNA repair.

Overall, functional category-based genetic stratification represents a molecularly informed, predictive diagnostic framework for assessing normal tissue radiosensitivity, complementing clinical and treatment factors, and may enable pre-treatment identification of patients at higher risk, thereby guiding personalized radiotherapy strategies to mitigate acute skin toxicity.

## Data Availability

Restrictions apply to the availability of these data. Data were obtained from Regina Maria Health Private Network and are available from the corresponding author with the permission of the aforementioned institution, due to privacy and ethical restrictions. Data sharing is subject to institutional approval and applicable data protection regulations.
